# INTERVENTIONS DURING MEALTIME FOR PEOPLE LIVING WITH DEMENTIA: A SCOPING REVIEW

**DOI:** 10.1093/geroni/igae098.0687

**Published:** 2024-12-31

**Authors:** Zih-Ling Wang, Basia Belza

**Affiliations:** University of Washington, Seattle, Washington, United States; University of Washington, Seattle, Washington, United States

## Abstract

Mealtime challenges among residents living with dementia in long-term care settings are a common problem. This study presents findings from a scoping review of mealtime interventions for individuals with dementia in long-term care settings. Using a Person-Environment-Occupation (PEO) Model, we categorize the effectiveness of all interventions to explore the holistic interplay of individual abilities, environmental conditions, and occupational sections in enhancing mealtime experiences for these individuals. From a total of 2,786 references identified through a three-step search strategy, 45 articles met the inclusion criteria, focusing on “eating,” “feeding,” “dementia,” and “mealtime” interventions from 2008 to 2023. The review highlights dementia-related eating challenges, such as using utensils improperly and consuming non-food items stemming from cognitive decline. Interventions span aromatherapy, music, visual and auditory aids, spaced retrieval training, the Montessori method, physical improvement programs, food service modifications, and individual assistance, targeting interpersonal, intrapersonal, and environmental transactions of eating. The PEO model categorizes these findings into person (behavioral interventions), environment (dining room layout and social interactions), and occupation (activities like the Montessori method and physical exercises). However, few interventions comprehensively address all three PEO components. This review provides insights for healthcare professionals, educators, and researchers on improving eating performance and independence in dementia care, emphasizing the need for future research to establish evidence-based protocols and implement them in care settings.

